# Development of a BALB/c 3T3 neutral red uptake cytotoxicity test using a mainstream cigarette smoke exposure system

**DOI:** 10.1186/1756-0500-7-367

**Published:** 2014-06-17

**Authors:** David Thorne, Joanne Kilford, Rebecca Payne, Linsey Haswell, Annette Dalrymple, Clive Meredith, Deborah Dillon

**Affiliations:** 1 British American Tobacco, Group R&D, Southampton, Hampshire SO15 8TL, UK; 2 Covance Laboratories Ltd, Otley Road, Harrogate, North Yorkshire HG3 1PY, UK

**Keywords:** Tobacco smoke, Whole smoke, Gas vapour phase, Smoke exposure system, Neutral red, BALB/c3T3

## Abstract

**Background:**

Tobacco smoke toxicity has traditionally been assessed using the particulate fraction under submerged culture conditions which omits the vapour phase elements from any subsequent analysis. Therefore, methodologies that assess the full interactions and complexities of tobacco smoke are required. Here we describe the adaption of a modified BALB/c 3T3 neutral red uptake (NRU) cytotoxicity test methodology, which is based on the Interagency Coordinating Committee on the Validation of Alternative Methods (ICCVAM) protocol for in vitro acute toxicity testing. The methodology described takes into account the synergies of both the particulate and vapour phase of tobacco smoke. This is of particular importance as both phases have been independently shown to induce in vitro cellular cytotoxicity.

**Findings:**

The findings from this study indicate that mainstream tobacco smoke and the gas vapour phase (GVP), generated using the Vitrocell® VC 10 smoke exposure system, have distinct and significantly different toxicity profiles. Within the system tested, mainstream tobacco smoke produced a dilution IC_50_ (dilution (L/min) at which 50% cytotoxicity is observed) of 6.02 L/min, whereas the GVP produced a dilution IC_50_ of 3.20 L/min. In addition, we also demonstrated significant dose-for-dose differences between mainstream cigarette smoke and the GVP fraction (P < 0.05). This demonstrates the importance of testing the entire tobacco smoke aerosol and not just the particulate fraction, as has been the historical preference.

**Conclusions:**

We have adapted the NRU methodology based on the ICCVAM protocol to capture the full interactions and complexities of tobacco smoke. This methodology could also be used to assess the performance of traditional cigarettes, blend and filter technologies, tobacco smoke fractions and individual test aerosols.

## Background

Routine in vitro toxicological assessment of tobacco smoke has been conducted through a variety of methods and tends to produce consistent responses. However, the majority of responses observed are based on the particulate fraction of cigarette smoke and not the complete smoke aerosol, which is comprised of both the particulate and vapour phase combined. This is partly because the particulate fraction of smoke can be captured with relative ease, whereas generating and exposing cells to a tobacco smoke aerosol is technically challenging and often requires specialised equipment. This is further compounded by the fact that there is over 30 years of testing the tobacco particulate fraction using standard, submerged culture methodologies. The testing of smoke particulate matter has generally been performed using several toxicological endpoints, such as the Neutral Red uptake assay (NRU), the in vitro micronucleus assay (IVMN), the Ames reverse mutation assay and the mouse lymphoma assay (MLA) [1-4]. These assays are consistent with many of the guidelines developed by the International Conference on Harmonization [5], the Committee on Mutagenicity [6] and, for tobacco smoke, Health Canada [7]. In addition, the Cooperation Centre for Scientific Research Relative to Tobacco (CORESTA) in vitro taskforce has also recommended a similar approach for analysis of tobacco products [8].

As tobacco smoke is a complex aerosol generally consisting of more than 6000 chemicals [9], distributed between both the vapour and particulate fractions, analysis of the particulate material only omits any interactions or responses generated by the vapour phase. This is particularly important as the vapour phase makes up the majority smoke fraction and contains known toxicants responsible for adverse health effects [10,11]. Furthermore, separating smoke fractions may lead to alterations or chemical changes which may not be representative of the complete smoke aerosol.

Over the last decade a great deal of focus has been placed on the development of tobacco mainstream smoke exposure systems [12-15], which capture the full interactions of both phases of tobacco smoke together and presents a more physicologically relevant test compound for the assessment of human risk.

The aim of this study was to utilise an adapted exposure methodology for the assessment of cigarette smoke, based on an existing NRU protocol for in vitro acute toxicity testing, developed by the Interagency Coordinating Committee on the Validation of Alternative Methods (ICCVAM) - NIH Publication no: 07–4519 [16]. Although the ICCVAM protocol is intended to be used with standard submerged cell cultures, we have modified it to assess the interactions of a mainstream tobacco smoke aerosol at the air-liquid interface (ALI) using BALB/c 3T3 cells.

ALI exposure ensures that cells are exposed to all components of the smoke aerosol, not just the soluble fraction, as would be the case under submerged conditions. The results from this study indicate that both the particulate and gas vapour phase (GVP) of tobacco smoke contribute significantly to smoke toxicity, based on the experimental set-up and parameters used. In addition to mainstream smoke exposure, the exposure system could potentially be further modified to deliver individual gases at the ALI, which could be used to support future in vitro testing scenarios. We further conclude this methodology could be used to assess the toxicity of existing and novel aerosol-based tobacco products, where traditional particulate exposure techniques may provide only limited information.

## Methods

### Chemicals and reagents

All chemicals and reagents were obtained from Sigma-Aldrich (Gillingham, UK) unless otherwise stated. All tissue culture media were obtained from Gibco® via Life Technologies (Paisley, UK).

### Reference cigarettes

Kentucky reference 3R4F cigarettes were obtained from the University of Kentucky (Kentucky, USA). Prior to smoking, cigarettes were conditioned for at least 48 hours and for no more than 10 days at 22 ± 1°C and 60 ± 3% relative humidity according to International Organisation of Standardisation (ISO - 3402:1999).

### Cell culture

Mouse fibroblasts (BALB/c 3T3 clone A31) were obtained from the European Collection of Cell Cultures. BALB/c 3T3 cells were maintained in Dulbecco’s Modified Eagle Medium (DMEM; containing 4 mM glutamine and 4.5 g/L glucose supplemented with 10% foetal calf serum (FCS) and penicillin/streptomycin) at 37 ± 1°C in an atmosphere of 5% CO_2_ in air. The use of FCS is a slight modification to the original protocol, however, previous work has demonstrated that Balb/c cells grow better in the presence of FCS compared to newborn calf serum (NCS). Penicillin/streptomycin was added to reduce the risk of contamination, which could be increased whilst under whole smoke exposure conditions.

For ALI exposure, monolayer cultures were prepared on 24 mm Transwells® (permeable membranes, Fisher Scientific, UK) by seeding 5×10^5^ cells in 1 mL DMEM into each Transwell® (pre-equilibrated by soaking in DMEM for at least 1 hour). 2 mL DMEM was also added to the well beneath each Transwell®. Cells were incubated for approximately 24 hours at 37 C in a humidified atmosphere of 5% CO_2_ in air to achieve 90-100% confluent monolayers. Near-confluent monolayers were used for exposure as the Transwell® membrane itself absorbs Neutral Red (NR) dye. Near-confluent monolayers reduce the uptake of NR by the membrane. As the exposure period is relatively short (in comparison to the cell doubling time) and endpoint determination is performed immediately after the exposure with no recovery, the high confluency at treatment was considered to have no adverse impact on the assay. Finally the exposure conditions in the module were not controlled for CO_2_, which could potentially result in an adverse pH change in the cell culture media during exposure. Therefore, to prevent extreme changes in pH, HEPES (25 mM) was added to the media placed beneath each Transwell in the exposure modules. pH analysis was conducted during initial protocol development and was shown not to change throughout the exposure period in either the smoke treated or untreated air control cultures (average pH of 7.69).

### Smoke generation and exposure

**Figure 1:**
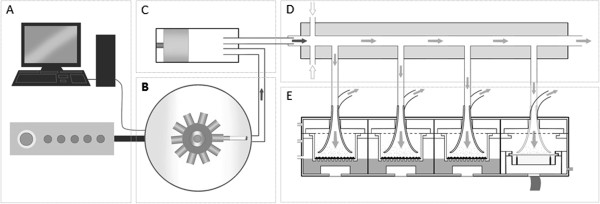
**A schematic representation of the Vitrocell® VC 10. [A]** Computer, software controller, which determines machine settings and smoking parameters. **[B]** Smoking Robot carousel where cigarettes are loaded and smoked. For GVP studies a Cambridge filter pad was installed into the line between the smoking carousel and the piston, for removal of the particulate smoke fraction. **[C]** Piston and syringe, which draws and delivers cigarette smoke to the dilution system. **[D]** Dilution of whole smoke occurs in the dilution bar. **[E]** Smoke exposure module which maintains cells at the ALI. To measure particulate dose, a quartz crystal microbalance was incorporated into the chamber, as shown, in position 4.

A Vitrocell® VC 10 Smoking Robot (Serial Number - VC10/090610) and 6/4 CF stainless steel exposure modules (Vitrocell® Systems, Waldkirch, Germany) were used to generate, dilute and deliver cigarette smoke to BALB/c 3T3 cells maintained at the ALI. The VC 10 is a rotary style smoking machine which has a single syringe that transfers the tobacco smoke to an independent continuous flow dilution system. The Vitrocell® dilution system uses both airflow (L/min) and vacuum rate (mL/min) to define the exposure concentration. Smoke dilution is first achieved via turbulent mixing in the dilution bar and different smoke concentrations are achieved by increasing or decreasing the diluting airflow. In addition to the diluting airflow, a vacuum sub-samples smoke (via negative pressure) from the dilution system into the module, which docks directly under the flow dilution system (Figure 1). The flow rate of the vacuum dictates the flow rate over the cells and was therefore maintained at 5.0 mL/min/well for all treatments.

Diluting air flow rates within this system were maintained using mass flow controllers (Analyt-MTC GmbH, Mülheim, Germany). Vacuum rates were set by mass flow meters (Analyt-MTC GmbH, Mülheim, Germany).

For each experiment, triplicate Transwells® were housed in a Vitrocell® 6/4 CF stainless steel module for exposure to freshly generated tobacco smoke from 3R4F cigarettes. Trumpet heights within the module were set at 2.0 mm above the Transwell® membrane. BALB/c 3T3 cells were exposed for 184 minutes on three independent occasions at the ALI to varied concentrations of either whole smoke or GVP. The GVP was generated by capturing the particulate material on a Cambridge filter pad positioned between the smoking head and piston. This enabled the cells to be exposed to the GVP without the associated particulate fraction. For all experiments, the VC 10 smoked to the ISO smoking regime (35 ml puff over 2 seconds, once a minute - ISO 4387:1991) using an 8 second exhaust.

### Neutral red uptake cytotoxicity test

The NRU cytotoxicity test performed was based on the ICCVAM BALB/c 3T3 test method protocol [16], with slight modifications. For our application, we used a whole smoke exposure system and exposed cells at the ALI rather than under standard submerged culture conditions as used in the ICCVAM protocol.

Following exposure (184 minutes) cells were incubated in DMEM culture media containing 50 μg/mL Neutral Red for 3 hours. Post-incubation, excess Neutral Red was washed off and intracellularly stored Neutral Red was released by the addition of Neutral Red de-stain solution (ethanol: acetic acid: distilled water; (50:1:49)). Neutral Red was measured by absorbance at 540 nm. Uptake of Neutral Red was determined for each treatment dilution and compared to that of air control cultures. For air control treatments, the diluting air flow rate was set at 0.2 L/min, and sub-sampled using a vacuum flow rate of 5.0 mL/min/well. As such, the flow rate over the cells was the same as that used for all smoke treatments. For each condition, the relative percentage cell survival and a dilution IC_50_ were calculated. The dilution IC_50_ was defined as a smoke dilution at which 50% cytotoxicity was achieved, based on a L/min diluting airflow rate.

A technical limitation of the VC 10 Smoking Robot is that under ISO smoking conditions it can only generate four doses and one air control. In order to obtain additional data points and calculate a more accurate IC_50_, we ran two exposures consisting of four doses per exposure, separated over two independent days per experiment. Using this approach we were able to expand the dose range tested and increase statistical power. As data were generated over different days, smoke-treatment data were compared to a concurrent air control, included in each exposure, thus providing the data with a daily base-line normalisation factor.

### Measurement of deposited particulate mass

To measure particulate deposition within the module during whole smoke exposure, one Quartz Crystal Microbalance - QCM (Vitrocell® Systems GmbH, Waldkirch, Germany) was installed into the last position of each 6/4 CF Stainless Steel Vitrocell® exposure module. QCM technology has previously been described in a set-up similar to this by Adamson et al., 2013 [17] and has been shown to correlate with particulate spectrofluorescence techniques. During the whole smoke generation and exposure phase, the QCM took mass readings every 2 seconds in real-time. Final deposited mass readings were only taken once the cigarette smoke had finished depositing onto the crystal, observed through a plateau phase in the real-time trace. QCMs in this study provided a valuable QC marker for smoke run consistency, and added confidence in the exposure approach, described above.

### Data presentation and statistics

Graphs were generated and analysed for a dilution IC_50_, and correlation coefficiencies using GraphPad Prism 6 (2012) statistical software, version 6.01. Microsoft Excel 2010 was used to generate the data tables, mean values and standard deviations. Statistical analysis was conducted using Minitab® version 16.1.0 using a 2-sample *T*-test and one-way analysis of variance (ANOVA). All assessments were conducted on at least three independent experimental occasions, with three replicates per occasion. Data were modelled using a sigmoidal four-parameter-logistic curve.

**Figure 2:**
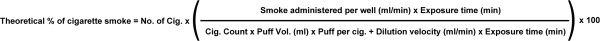
**Equation for calculating theoretical smoke dose [**[18]**].**

Theoretical percentage of cigarette smoke was calculated from Webber et al.,2013 [18] using the following equation (Figure 2).

Nicotine equivalents were back calculated using theoretical percentage smoke calculations and a measured starting nicotine concentration of 0.7 mg/cigarette [19].

## Results

A summary of all the data obtained for whole smoke and GVP can be found in Table 1, which includes percentage theoretical smoke exposure, theoretical nicotine exposure (mg) and calculated IC_50_ concentrations.

**Table 1:** Summary of whole smoke and GVP cytotoxicity results

**Airflow (L/min)**	**Log**_**10 **_**airflow**	**Theoretical% smoke exposure**	**Theoretical nicotine exposure (mg)**	**Whole smoke**				**Gas vapour phase**			
				**% relative survival ± SD**	**Dilution IC**_**50 **_**(L/min)**	**% smoke IC**_**50**_	**Theoretical nicotine exposure IC**_**50 **_**(mg)**	**% Relative survival ± SD**	**Dilution IC**_**50 **_**(L/min)**	**% smoke IC**_**50**_	**Theoretical nicotine exposure IC**_**50 **_**(mg)**
10.00	1.00	1.195	0.008	89.42 ± 13.22	6.02	1.99	0.014	100.22 ± 2.99	3.20	4.15	0.029
8.00	0.90	1.493	0.010	70.89 ± 25.21				101.43 ± 2.14			
7.00	0.85	1.705	0.012	65.06 ± 15.38				90.97 ± 7.66			
6.00*	0.78	1.987	0.014	46.90 ± 4.08				98.87 ± 4.33			
5.00*	0.70	2.382	0.018	27.14 ± 11.91				75.57 ± 10.53			
4.00*	0.60	2.973	0.021	4.08 ± 5.10				84.82 ± 8.07			
3.00*	0.48	3.952	0.028	3.00 ± 8.60				44.38 ± 12.42			
2.50	0.40	4.731	0.033	~				21.40 ± 6.58			
2.00	0.30	5.892	0.041	~				12.13 ± 11.04			
1.00	0.10	11.577	0.080	0.83 ± 7.28				8.65 ± 7.23			

Statistical difference denoted by*.

**Figure 3:**
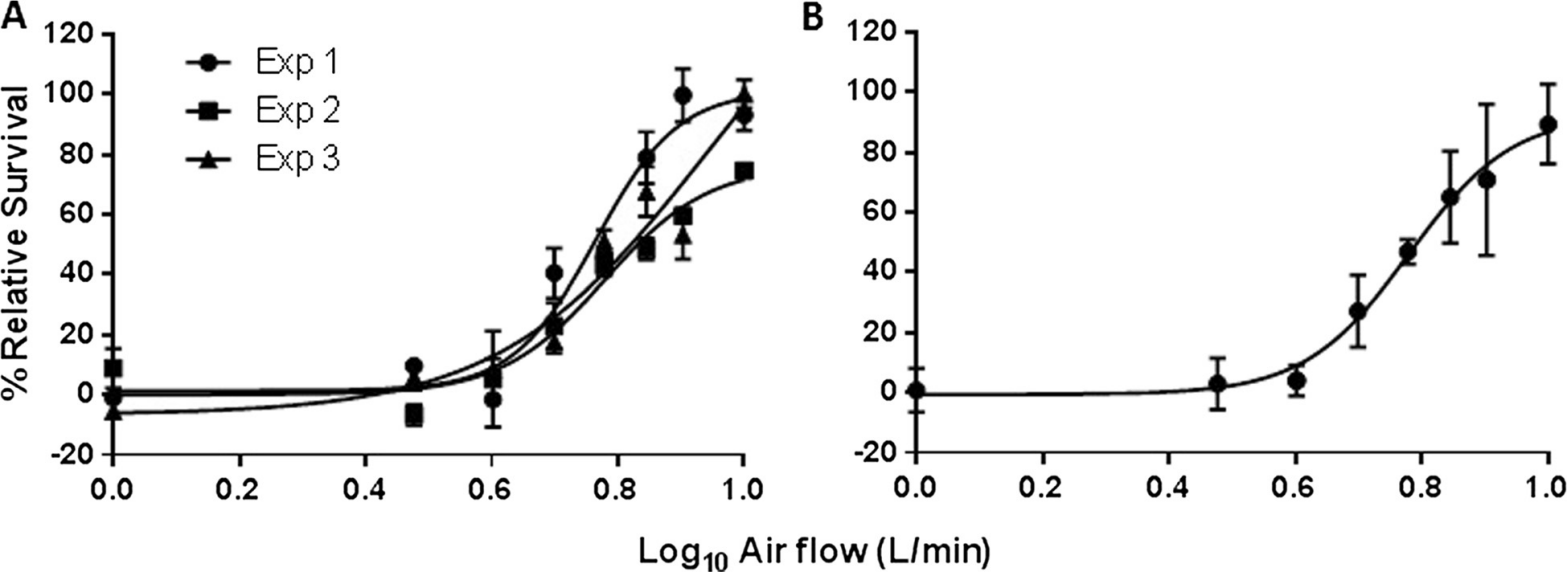
**Whole smoke dose–response. [A]** Percentage relative cell survival from three independent experiments, indicating inter- and intra-experimental variation. **[B]** Mean pooled data from three experiments with a correlation coefficient of R^2^ = 0.90 and a dilution IC_50_ of approximately 6.02 L/min for the 184 minute exposure to mainstream tobacco smoke.

Using the Vitrocell® VC 10 Smoking Robot and exposure system, we were able to expose BALB/c 3T3 cells to freshly generated whole smoke or the GVP and measure cytotoxic responses over a 3 hour exposure period (184 minute exposure, 23 cigarettes delivered at 8 puffs per cigarette). Results from whole smoke exposure across airflows of 1.0-10.0 L/min gave a dilution IC_50_ of 6.02 L/min with relative percentage survival ranging from 100 – 0 percent viability, when compared against the concurrent air control. Based on three independent experiments we demonstrated a correlation coefficient of R^2^ = 0.90 and a consistent dose–response. We also demonstrated that smoke dilutions of 1.0, 3.0, 4.0, 5.0, 6.0, 7.0, 8.0 and 10.0 L/min correspond with an average relative cell survival of 0.83 ± 7.28, 3.0 ± 8.59, 4.08 ± 5.10, 27.14 ± 11.91, 46.90 ± 4.08, 65.06 ± 15.39, 70.89 ± 25.21, 89.42 ± 13.22 percent respectively (Figure 3).

**Figure 4:**
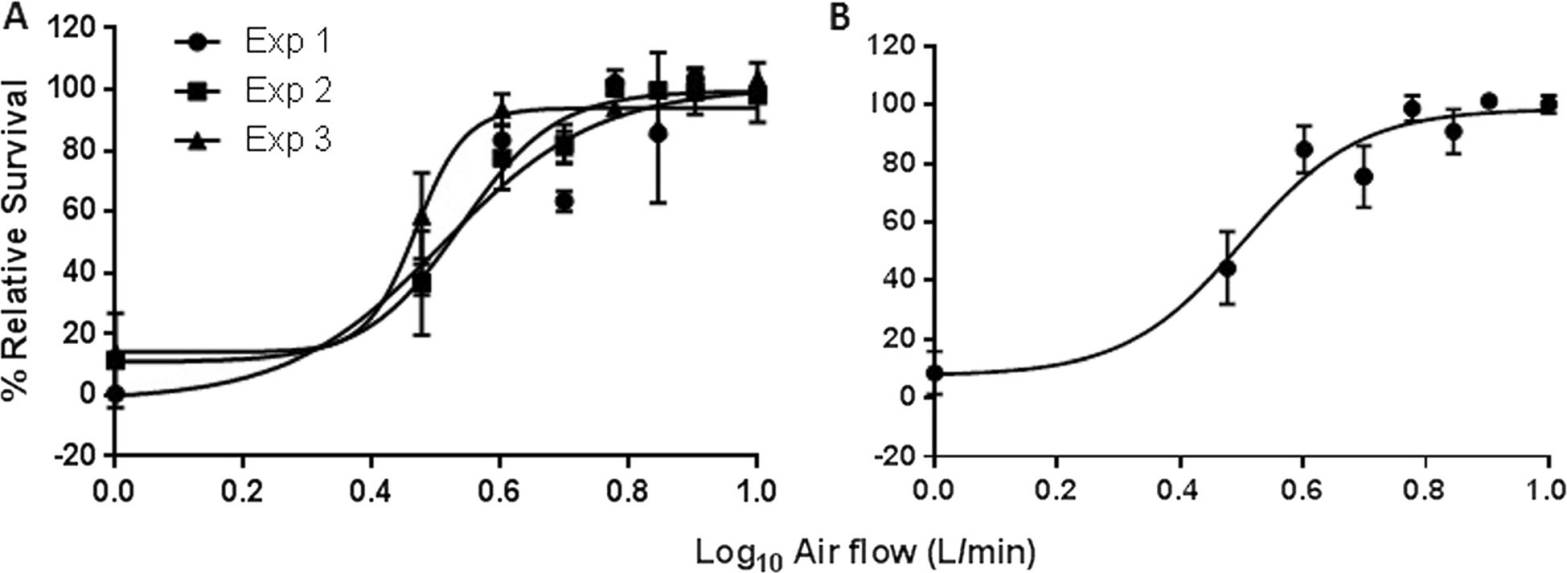
**Gas vapour phase dose–response. [A]** Percentage relative cell survival from three independent experiments, indicating inter- and intra-experimental variation. **[B]** Mean pooled data from three experiments with a dilution IC_50_ of approximately 3.2 L/min and a correlation coefficient of R^2^ = 0.92 for the 184 minute exposure to the gas vapour phase of tobacco smoke using 3R4F cigarettes.

The GVP data also showed a consistent dose–response between experiments, generating an average dilution IC_50_ of 3.20 L/min over a 184 minute exposure period. Based on three independent experiments, we demonstrated that GVP smoke dilutions of 1.0, 3.0, 4.0, 5.0, 6.0, 7.0, 8.0 and 10.0 L/min correspond with an average relative cell survival of 8.65 ± 7.23, 44.38 ± 12.42, 84.82 ± 8.07, 75.57 ± 10.53, 98.87 ± 4.33, 90.97 ± 7.66, 101.43 ± 2.14, 100.22 ± 2.99 percent respectively. We also observed an average correlation coefficient fit of R^2^ = 0.92 for the three independent experiments (Figure 4).

**Figure 5:**
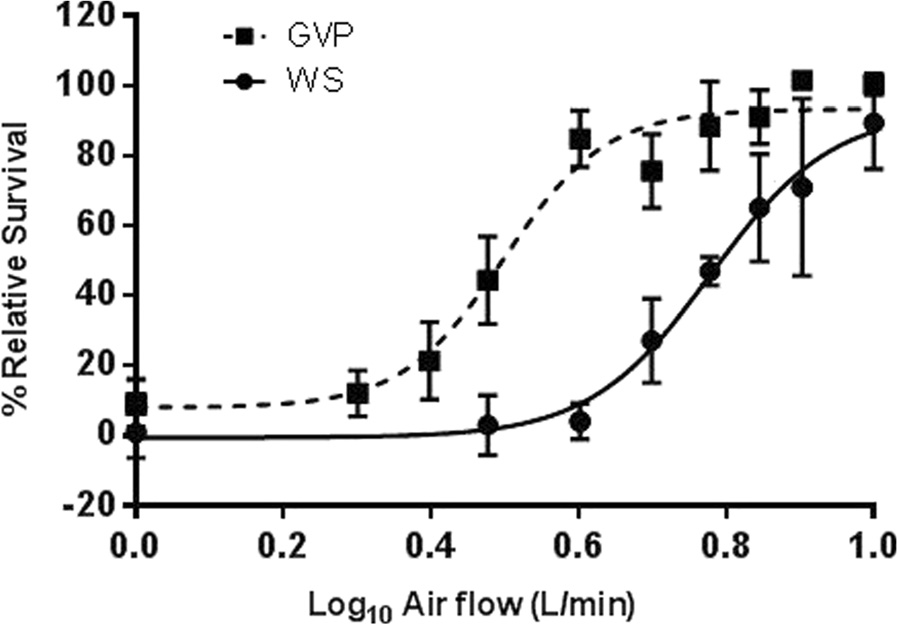
A comparison between the cytotoxicity of mainstream tobacco smoke (WS) and the gas vapour phase (GVP).

When whole smoke was compared to the GVP, the GVP showed significantly less cytotoxicity and variability, producing a dilution IC_50_ of 3.20 L/min compared to 6.02 L/min. This indicates that in this system and under this experimental set-up, both the GVP and particulate fractions, or an interaction between the two, are responsible for smoke toxicity. When comparing whole smoke and GVP, at equivalent airflows, there are clear statistical differences at 3.0, 4.0, 5.0 and 6.0 L/min with P-values of 0.018, 0.001, 0.013, 0.001 respectively. The remaining airflows of 1.0, 7.0, 8.0 and 10.0 L/min showed no statistical difference between whole cigarette smoke and the GVP fraction, which is unsurprising given either the complete toxicity or complete relative survival observed (Figure 5). Given the difference in cytotoxicity between the two smoke phases, a narrowed dose range experiment was conducted on the GVP fraction alone, using higher smoke concentrations. This additional set of experiments at 1.0, 2.0, 2.5 and 6.0 L/min were conducted in accordance with the developed protocol and compared to concurrent air controls obtained on the same day. Data was combined with previously obtained data and is presented in Figure 5 and Table 1. The observed differences in cytotoxicity is compounded by the higher level of variation in cell survival observed between whole smoke compared to the GVP exposures. This variation could be attributed to the particulate phase of smoke or an interaction between the two phases, but without a more in-depth investigation, the variation observed between the exposures cannot be clearly defined.

In order to provide a tangible measure between smoke exposures and to assess smoke run consistency, a QCM acted as a Quality Control (QC) marker for smoke exposure, measuring deposited mass in a real-time in situ format. Table 2 shows the absolute total deposited mass values obtained for all whole smoke exposure experiments and mean and standard deviation of the data. These data confirm that deposited mass (μg/cm^2^) readings between experiments were consistent, giving confidence in the exposure set-up and experimental conditions.

**Table 2:** Summary of deposited mass results obtained in situ of whole smoke exposure using QCM technology

**Airflow (L/min)**	**Deposition (μg/cm**^**2**^**)**	**Mean deposited mass ± SD (μg/cm**^**2**^**)**
1.0	30.11	27.50 ± 2.26
	26.06	
	26.34	
3.0	8.13	7.42 ± 0.62
	7.02	
	7.10	
4.0	4.18	4.30 ± 0.22
	4.56	
	4.17	
5.0	3.16	2.99 ± 0.17
	2.82	
	2.98	
6.0	1.90	1.83 ± 0.13
	1.92	
	1.69	
7.0	1.11	1.13 ± 0.25
	1.38	
	0.89	
8.0	0.68	0.63 ± 0.10
	0.69	
	0.52	
10.0	0.32	0.28 ± 0.05
	0.30	
	0.22	

## Findings

In this study, we adapted the ICCVAM protocol for in vitro acute toxicity testing for measurement of the toxicity of cigarette smoke fractions at the ALI, which is particularly important as different smoke fractions have independently been shown to induce cellular cytotoxicity [15,20]. We further demonstrate that BALB/c cells are compatible at the ALI and remain viable for at least 184 minutes under flowing air conditions (5 mL/min/well).

Using this modified protocol we have generated tobacco smoke cytotoxicity data ranging from 100 – 0% relative viability compared to concurrent air controls. Furthermore, by selectively filtering the particulate phase on a Cambridge filter-pad we have also assessed the cytotoxicity of the GVP independently to the whole smoke aerosol. These data indicate that both whole smoke and the GVP play a role in tobacco smoke cytotoxicity in this system and that both have significantly different toxicity profiles, as demonstrated by dilution IC_50_ values of 6.02 and 3.20 L/min, respectively. This protocol further allows for the assessment of semi-volatiles and vapour phase compounds where the ICCVAM test method protocol is limited to soluble and ‘some volatile’ compounds. It states, “although this test method is not suitable for highly volatile substances, mildly volatile substances may be tested with some success” [16]. Volatile test substances may generate vapours in submerged culture conditions which could become reabsorbed into the treatment medium in adjacent wells, causing cross-contamination, resulting in inaccurate data. In our experimental set-up, cross-well contamination cannot occur as each well is independent from the next due to the structure of the exposure module. Although we describe a method developed with tobacco smoke at the ALI, this set-up may also lend itself to the assessment of individual gaseous components of cigarette smoke that are potential candidates for the adverse health effects associated with tobacco smoking. For example, many of the aldehydes have known toxicological properties and are volatile in solution, making the assessment of these particularly difficult in vitro*,* especially under submerged culture conditions [21].

The cytotoxicity of whole mainstream tobacco smoke and its GVP have been assessed in a variety of other studies using ALI exposure technologies [15,22-26]. In these studies the GVP fraction was found to have an independent toxicity profile. Our study confirms these findings and, along with other cigarette smoke vapour phase studies, highlights the importance of using an appropriate exposure system capable of exposing cells to both fractions of cigarette smoke.

The Vitrocell® VC 10 exposure system described in this study does have a limitation. The system can only generate four doses in one exposure run, based on ISO smoking conditions. We have demonstrated here that pooling data from two exposures provides a viable solution to this problem. However, there are limitations to this approach that need careful consideration and further investigation. For example, this study has not investigated day-to-day or exposure-to-exposure biological variability. For this, further work needs to be conducted. In addition, there may be ways in which to modify the exposure set-up to produce additional doses that this study has not investigated. By using dose tools, such as QCM technology, we have shown that we can link smoke runs together and can demonstrate smoke run consistency. QCMs only work, however, where there is particle deposition to be measured. For GVP studies, where there is no deposited mass, QCMs alone will not suffice. Therefore, potential vapour-phase dose tools are required to support future cigarette smoke assessment [27].

Finally, data has been tabulated as a function of theoretical percentage smoke exposure and theoretical nicotine exposure (mg) with respective IC_50_ calculations. As yet there is no consensus on how to present whole smoke data and by presenting data in this format, it allows others to consider these data and make appropriate comparisons. By presenting data primarily as a function of diluting airflow (L/min), we believe we have presented it in its simplest form, which ultimately avoids assumptions and misinterpretation of data. Analysis of smoke delivery and exposure may, in the future, define a more accurate way to present whole smoke data.

Based on observed responses from this study, we propose that this system can be used to assess conventional tobacco products as well as other aerosols and gases. It could be especially beneficial when assessing modified tobacco products e.g. those that contain filter modifications aimed at reducing vapour phase-based smoke toxicants [26,28]. With traditional exposure techniques the analysis of these cigarettes would be limited as the vapour phase would not be captured for biological analysis. In addition to these vapour phase-based filter modifications, there is capacity to modify the tobacco blend for the removal of particulate-based toxicants such as tobacco specific nitrosamines (TSNAs) [29]. An exposure system that can be used to compare modified tobacco products and capture the true particulate-vapour-phase interactions would be extremely useful to the tobacco industry and other parties involved in aerosol-based research.

## Abbreviations

ALI: Air-liquid interface; DMEM: Dulbecco’s modified eagle’s medium; FCS: Foetal calf serum; GVP: Gas vapour phase; IARC: International Agency for Research on Cancer; ICCVAM: Interagency Coordinating Committee on the Validation of Alternative Methods; Dilution IC_50_: Diluting airflow at which 50% cytotoxicity is observed; ISO: International Standards Organization; IVMN: In vitro micronucleus assay; MLA: Mouse lymphoma assay; NCS: Newborn calf serum; NRU: Neutral red uptake assay; QC: Quality control; QCM: Quartz crystal microbalance; SD: Standard deviation; VC 10: Vitrocell® VC 10 Smoking Robot; WS: Whole smoke.

## Competing interests

The authors declare there are no competing interests and are employees of British American Tobacco or contracted by British American Tobacco. Covance Laboratories Ltd, Harrogate, UK, conducted all experimental work, which was funded by British American Tobacco.

## Authors’ contributions

JK and RP conducted all experimental work and provided scientific support. DT wrote the manuscript and provided scientific direction. LH conducted all graphical and statistical analysis, whilst AD, CM and DD scientifically reviewed the experimental design and concept and provided scientific support. All authors approved the final manuscript.
